# The Judgments and Emotion Attributions in Peer Exclusion Situations among Fourth, Sixth, and Eighth Graders in Japan

**DOI:** 10.3390/ijerph20075306

**Published:** 2023-03-29

**Authors:** Mari Hasegawa

**Affiliations:** Graduate School of Education, Tohoku University, Sendai 980-8576, Japan; mari.hasegawa.c2@tohoku.ac.jp

**Keywords:** bullying, peer exclusion, bystander, emotion attribution, judgments, emotions

## Abstract

This study examined the judgments and emotion attributions in peer exclusion situations among Japanese middle-childhood children (fourth graders and sixth graders) and adolescents (eighth graders). In total, 371 participants were presented with one of three bystander conditions—no bystander, passive bystander, or active bystander—and asked to judge the excluders’ behavior and attribute emotions toward excluders. Here, excluders are children who physically or emotionally separate other children from social groups. All scenarios involved a child wishing to join a peer group but was rejected (that is, excluded from the group), and there were three types of situations: one in which there were no bystanders, one in which the bystanders did not respond, and one in which the bystanders allowed the excluded child into their group. The excluded target was presented as either violent or shy. Furthermore, the participants assessed their own bullying and bystander behaviors in their daily lives. Adolescents judged excluders as less immoral and as having positive emotions more often than did children. Both children and adolescents judged the exclusion of violent targets to be less serious than the exclusion of shy targets. There were no differences in judgments and attributions according to bystander types. There was weak evidence of a relationship between self-reported bullying/bystander behavior, and judgment in fictitious settings was obtained.

## 1. Introduction

Bullying has been defined as “be[ing] exposed, repeatedly and over time, to negative actions on the part of one or more students” [1]. Bullying is a universal problem occurring in most schools across different cultures [2]. There are various types of bullying, such as physical and verbal attacks, cyberbullying, and peer exclusion [3]. Peer exclusion is defined as the experience of being physically or emotionally separated from others [4]. Such social exclusion has deleterious consequences because the victims are left with no support from the peer group that would have helped them manage rejection experienced via other types of bullying [5].

A large body of research exists on children’s and adolescents’ judgments regarding peer exclusion. In recent decades, this social-cognitive research on social exclusion has been integrated with research on children’s and adolescents’ emotional attributions following peer exclusion. The major focus has been on emotion attribution, especially the “happy victimizer” (HV) effect, a phenomenon in which the perpetrator is assumed to have positive emotions [6,7]. Emotion attribution is related to social behavior [6]. Specifically, the HV response was found to have a negative correlation with prosocial behaviors and a positive correlation with aggressive behaviors [8]. The HV phenomenon is generally more prevalent in early childhood and decreases in middle childhood. However, in peer exclusion situations, it was shown to increase after middle childhood and into adolescence [9]. As studies have mostly been conducted in Europe and the United States, it is important to examine whether the same results can be obtained in Japan. The purpose of this study is to explore grade-level differences in judgments and emotional attribution regarding peer exclusions.

Bullying in schools is conceptualized as a group process [10,11,12], in which all students in a class are somehow involved in or aware of, even if they do not actively participate in, acts of bullying [13]. Bystanders who were neither perpetrators nor victims were present in more than 80% of bullying situations [14,15]. Mulvey et al. [3] also indicated the role of bystanders in situations of peer exclusion. Bystanders have different behavioral choices, including protecting the victim, reinforcing the bullying (by laughing, watching, or other responses), participating in the bullying, keeping their distance as an outsider, or leaving the scene [3,16]. The present study distinguished between active bystanders, who stand up for the victim and intervene to stop the bullying, and passive bystanders, who ignore the bullying or pretend not to notice, as the presence of active bystanders can prevent bullying [17].

Some studies [13,18] have indicated that bullies recognize that passive bystanders tacitly approve of their behavior. In other words, bystanders support bullying by not intervening [19]. Therefore, the judgment regarding bullying in a group can differ based on the types of bystanders. It is not clear, however, whether children believe that passive bystanders affirm bullying or that exclusion is more acceptable when passive bystanders are present. The second purpose of this study is to examine how judgments and emotions related to peer exclusion vary by the presence of passive and active bystanders.

In a study that examined the impact of bystanders on judgment and emotions regarding peer exclusion, Malti et al. [16] showed three types of bullying videos to participants aged 12 and 16 years: no bystanders, onlooking bystanders (observing and not intervening), and inclusive bystanders (accepting the excluded target into their group). The participants judged onlooking bystanders as more wrong than inclusive bystanders. Furthermore, both age groups attributed more positive emotions to the excluders in the presence of bystanders compared to situations in which no bystanders were present. These results suggest that the presence of bystanders might change peer exclusion judgments and that the judgments differ according to the children’s developmental stage.

Malti et al. [16] reported significant findings suggesting that bystanders’ behavior affects judgments related to peer exclusion. We sought to expand these findings and examined the period from middle childhood to early adolescence when bullying increases and bystander intervention behaviors decrease [20]. Similar to Malti et al. [16], we evaluated middle childhood peer exclusion based on the presence and types of bystanders.

In addition, participants may consider not only group but also target characteristics in their exclusion decisions. One factor that justifies exclusion is whether it conflicts with moral issues. Thus, if the target acts immorally or if the target has behavioral tendencies unrelated to morality, participants may be more likely to approve of excluding the first target. The third purpose of this study was to determine whether different types of targets produce differences in judgments and attributions regarding peer exclusion. This study presented two scenarios: a person with a violent personality who was excluded and a shy person who was excluded.

As previously mentioned, studies in Europe and the United States have shown that children tend to attribute negative emotions to the perpetrators of peer exclusion, but adolescents tend to attribute positive emotions to the perpetrators. Similar results were expected from this study on Japanese children and adolescents.

Thus, this study examined the effects of active and passive bystanders on judgment and emotion attribution in peer exclusion settings in middle childhood and early adolescence. The following five hypotheses were investigated:

**Hypothesis 1.**  *Middle-childhood children are more likely to judge excluders as immoral and attribute negative emotions to them than adolescents*.

**Hypothesis 2.**  *Participants judge excluders in the passive bystander condition as less immoral and attribute more positive emotions to them than excluders in the no-bystander or active-bystander condition*.

It is expected that judgments regarding exclusion will differ depending on whether the behavioral characteristics of the target of exclusion are immoral (e.g., violent behavior) or based on personal characteristics (e.g., shy).

**Hypothesis 3.**  *Participants are more likely to judge excluders as immoral and attribute negative emotions to them in the shy target situation than in the violent target situation*.

Furthermore, we examined the correlations between self-reported bullying and bystander behavior, and judgment and emotion attribution. Again, moral judgments are related to social behavior; therefore, it is reasonable to expect in this study that judgment will be related to bullying behavior and bullying bystander behavior. Some researchers have argued that emotion attribution and prosocial and antisocial behaviors are correlated (for review, see [21]). Hence, we predicted that judgments and emotion attribution would be correlated with bullying and bystander behaviors in peer exclusion situations.

**Hypothesis 4.**  *The more people report bullying behavior, the more likely they are to judge excluders as less immoral and attribute more positive emotions to them*.

**Hypothesis 5.**  *The more people report passive bystander behavior, the more likely they are to judge excluders as less immoral and attribute more positive emotions to them*.

## 2. Materials and Methods

### 2.1. Participants

Japanese children and adolescents (*N* = 431) in the fourth grade (M = 9 years, 6 months, *n* = 130), sixth grade (M = 11 years, 6 months, *n* = 163), and eighth grade (M = 13 years, 7 months, *n* = 138) participated in this study. Participants with inappropriate responses to a question about lying and those who did not give complete responses (*n* = 60) were excluded from the study. Thus, the data of 371 participants in the 110 fourth grade (M = 9 years, 6 months, 56 boys, and 54 girls), 143 in the sixth grade (M = 11 years, 6 months, 69 boys, and 74 girls), and 118 in the eighth grade (M = 13 years, 7 months, 69 boys, and 49 girls) were analyzed. All participants were from middle-income families living in metropolitan areas, with Japanese as their native language. The permanent ethics committee of Yokohama City University approved the study design [Ethic Committee Name: Yokohama City University Ethics Committee, Approval Code: H-2018-2, Approval Date: 26 September 2018].

### 2.2. Measures

We presented the following questions in a booklet that consisted of four pages.

#### 2.2.1. Judgment Related to Peer Exclusion

To decide on the fictional scenarios and characters used for this study, we discussed (in advance) with elementary and junior high school teachers the ethical issues related to each scenario. The foundation of each scenario includes a character who approaches a group and says, “I want to play together,” but the character is rejected. We prepared six scenarios: three conditions (no bystanders, passive bystanders, and active bystanders) × two excluded target types (violent and shy [Appendix A]). Each condition was a between-subjects factor, and the type of excluded target was a within-subjects factor. The type of excluded target was presented in a randomized order on the class level for children and individually for adolescents. We gave unisex Japanese names to the characters, all of whom were in the same grade as the participants. Participants responded to the following question after listening to two types of stories regarding their judgment of the exclusion: “How ‘okay’ was it for the group not to allow the excluded target to join them?” Participants’ responses were rated on a 4-point Likert-type scale ranging from 1 = okay to 4 = not okay.

#### 2.2.2. Emotion Attribution

One item referred to attributing emotion to the excluders: “How did the excluders in the group feel after they did not let the target join them?” Participants’ responses to this question were rated on a 4-point Likert-type scale ranging from 1 = happy to 4 = sad.

#### 2.2.3. Bullying Behavior Scale

We used the bullying behavior scale developed by Okayasu and Takayama [22], which includes three statements: “I excluded, ignored, or spoke ill of someone with my friends”; “I harassed or tricked someone (scribbling, hiding things, etc.) with my friends”; and “I deliberately bumped into, hit by pretending to play, or kicked someone with my friends.” These questions pertained to activities from the previous six months. Participants’ responses were rated on a 5-point Likert-type scale ranging from 0 = never to 4 = often. We calculated the mean score of each participant for the analysis.

#### 2.2.4. Bystander Behavior Scale

We used the passive bystander behavior scale based on Okayasu and Takayama [22] with minor modifications by Nishino [23], which includes three statements: “I was just observing someone being deliberately bumped into, being hit while pretending to play or kicked”; “I was just observing when someone’s things were hidden or scribbled on”; and “I was just observing when someone was excluded from a group, ignored, or spoken ill of.” These questions covered the previous six months. Participants’ responses were rated on a 5-point Likert-type scale ranging from 0 = never to 4 = often. We calculated the mean score of each participant for the analysis.

#### 2.2.5. Social Desirability Item

Self-reports of bullying behavior may be underestimated. Therefore, to eliminate participants who answer based on social desirability, we also included the item “I lied to a friend or a family member” in a series of statements regarding bullying behaviors. Participants’ responses were rated on a 5-point Likert-type scale ranging from 0 = never to 4 = often. Participants who selected “never” were excluded from the analysis.

### 2.3. Procedure

The survey was conducted between October and December 2019. Classroom teachers distributed and collected the questionnaires during class. The teachers read all of the statements aloud to the middle-childhood children, and the students responded immediately. Adolescents read the booklets containing the research questions themselves, and the students responded at their own pace. Three types of bystander conditions were allocated to each elementary class, whereas the types of bystander conditions were randomly distributed to the adolescents. Classroom teachers explained the ethical considerations, including that participation was voluntary and that individuals would not be identified, and requested the participants’ assent. In addition, the parents of the children were given a written explanation of the ethical issues by the principal and provided their consent.

## 3. Results

Table 1 shows the means and standard deviations for the judgment and emotion attribution scores depending on the school grade and condition. Table 2 shows the means and standard deviations of bullying behavior and bystander behavior scores depending on the school grade.

### 3.1. Test for Hypotheses 1, 2, and 3

All of the following analyses were performed in SPSS ver. 26 (IBM, Tokyo, Japan). Prior to testing the hypotheses, a four-factor analysis of variance was conducted to examine the effect of gender: three school grades (fourth, sixth, and eighth grade) × three conditions (no bystander, passive bystander, and active) × two targets (violent and shy) × gender. The results indicated that only the interaction of school grades × conditions × gender on the emotion attribution scores was significant (F(4346) = 3.37, *p* < 0.05, partial η^2^ = 0.04)).

The results of an ANOVA for three school grades (fourth, sixth, and eighth grade) × three conditions (no bystander, passive bystander, and active bystander) × two targets (violent and shy) on the judgment scores indicated that the main effects of the grades and targets were significant; F(2360) = 19.74, *p* < 0.001, partial η^2^ = 0.10, and F(1360) = 125.33, *p* < 0.001, partial η^2^ = 0.26 (Table 1). Furthermore, the grades × targets interaction was significant; F(2360) = 8.16, *p* < 0.001, partial η^2^ = 0.04. Post-hoc comparisons using the Bonferroni test (*p* < 0.05) indicated that the difference in scores across the two types of targets per grade was not significant, and the difference in scores across the three grades per target was not significant.

Furthermore, the results of an ANOVA for three school grades (fourth, sixth, and eighth grade) × three conditions (no bystander, passive bystander, and active bystander) × two targets (violent and shy) on the emotion attribution scores indicated that the main effects of grades were significant; F(2355) = 7.39, *p* < 0.001, partial η2 = 0.04 (Table 1). Post-hoc comparisons using the Bonferroni test (*p* < 0.05) indicated that eighth-grade participants attributed “happy” feelings to the excluders more frequently than did other grades.

As mentioned above, because the interaction of grades × conditions × gender on the emotion attribution scores was significant, post-hoc comparisons were made using the Bonferroni test (*p* < 0.05). The results indicated that the conditions × gender interactions on fourth and sixth graders were not significant, and female eighth graders were more likely than male eighth graders to attribute “happy” feelings to the excluders in the active bystander condition.

### 3.2. Test for Hypothesis 4: Relationship between Bullying Behavior, Judgment, and Emotion Attribution

To test our hypotheses regarding the predictive effects of judgments and emotion attribution on bullying behaviors, a hierarchical multiple regression analysis was performed using the scores for bullying behaviors as the dependent variable. Although the gender effect was not included in the hypotheses, it was included in the analysis as a controlling factor because gender differences in social behavior were often observed [24]. We entered the child’s age group, gender, and three conditions (each converted to binary data of 1 or 0) as the control variables in step 1 of the regression model. Judgment scores were entered in step 2, and emotion attribution scores were entered in step 3.

Results indicated that bullying behavior was predicted by the child’s gender and judgment toward a violent target (Table 3).

### 3.3. Test for Hypothesis 5: Relationship between Bystander Behavior, Judgment, and Emotion Attribution

To test our hypotheses regarding the predictive effects of judgments and emotion attribution on bystander behaviors, a hierarchical multiple regression analysis was performed using the scores for bystander behaviors as the dependent variable. We entered the child’s age group, gender, and three conditions (each converted to binary data in the form of 1 or 0) as the control variables in step 1 of the regression model. Judgment scores were entered in step 2, and emotion attribution scores were entered in step 3.

The results indicated that bullying behavior was predicted by judgment toward a violent target (Table 4).

## 4. Discussion

This study investigated how middle-childhood children and adolescents judged peer exclusion situations and whether their judgments were correlated with self-reported bullying and bystander behavior. The results indicated a significant main effect of school grade on the evaluation of excluders’ undesirability, with adolescents making more positive judgments related to peer exclusion than middle-childhood children. However, the differences in the interactions between the school grades and conditions were not significant. Similarly, the emotional attribution of the excluders showed a main effect of grades, with adolescents attributing happier feelings to the excluders than middle-childhood children. This result is consistent with previous studies showing the emergence of the HV phenomenon in peer exclusion situations (e.g., Malti et al. [9]). However, because there was no interaction between grade and condition, nor was there a main effect of condition, participants—regardless of age group—did not make judgments based on the type of bystander. Thus, Hypothesis 1 was supported, but Hypothesis 2 was not.

Next, this study presented two types of excluded targets: one with a violent personality and one with a shy personality. In social domain theory [25], violence is included in the moral domain, and a shy personality is included in the personal domain. The exclusion of targets for morally bad behavior was assumed to be more acceptable. Overall, the exclusion of the violent target was approved of more often than that of the shy target. Thus, Hypothesis 3 was supported.

There was little effect of gender on judgments and emotion attributions. In the active bystander condition, female eighth graders attributed more positive emotions to the excluders than male eighth graders. Since adolescent girls tend to desire more harmonic relationships [26], it is likely that a peaceful resolution in which the excluded person is accepted by another group will elicit positive emotions. However, further research is needed to confirm the correctness of this prediction.

Finally, the results of this study suggest that self-reported bullying behavior correlated with judgment and emotion attribution in fictitious settings, and judging the morality of peer exclusion toward the violent targets was associated with lower self-reported bullying behavior. Thus, Hypotheses 4 and 5 were partially supported. However, the statistical values were very small and should be considered weak evidence. It should be noted that the low average score of bullying/bystander behavior occured possibly because the “frequency” of bullying was assessed in this study. Participants may not have falsely reported that they did not bully, but they may have understated the actual frequency of bullying behavior.

The present study is significant in that the results are the same as those suggested in Western studies, which suggests that adolescents tend to attribute happy rather than sad feelings to excluders. The results also suggest that even elementary school students can make judgments based on target characteristics. Contrary to expectations, however, their judgments did not change according to the types of bystanders. There are two possible reasons for the failure to replicate the previous study’s finding that judgments differ depending on the type of bystander [16]. The first possibility is that differences in the way the stories were presented affected the results. Unlike the previous study, which presented a video, this study presented written text. Possibly, the video had a greater impact on the viewer. The second possibility is that the subjects in this study were younger than in previous studies. According to Selman [27], the integration of third-party perspectives is still difficult for elementary school children to comprehend.

The limitations of the present study and future issues include the lack of clarity regarding cognitive factors causing differences based on the school grade, as indicated in this study. It is necessary to conduct studies using an experimental design to identify the cognitive functions correlated with judgment and emotion attribution. Moreover, the current findings were obtained in a specific situation. The excluded targets were presented as children with typical personalities who tend to be targeted for bullying. In studies conducted in Europe and the United States, race-related issues have been more focused on than the personality traits of the victim [28]. The extent to which the present study’s findings are generalizable needs to be examined by conducting studies using both personality and ethnicity as the excluded targets. Finally, participants in this study self-evaluated bullying and bystander behavior. The extent to which participants can accurately evaluate their own behavior is unclear; it is suggested that evaluations made by teachers and peers should be utilized in future studies. Nevertheless, the present study is significant from a theoretical and educational perspective because it examined judgment related to peer exclusion in the presence of bystanders, which has not been adequately investigated to date. Future studies on judgment and emotion attribution in bullying situations should be conducted with samples that are more diverse in terms of age, race, and setting.

## 5. Conclusions

This study investigated how middle-childhood children and adolescents judged peer exclusion situations and whether their judgments correlated with self-reported bullying and bystander behavior. The results indicated that (1) adolescents judged excluders’ behavior as less immoral and attributed more positive emotion to them than children did; (2) there were no differences in judgments or attributions according to bystander types; (3) both children and adolescents judged exclusion toward violent targets to be less serious than exclusion toward shy targets; and (4) weak evidence of a relationship between self-reported bullying and bystander behavior and judgment in fictitious settings was obtained. This finding (i.e., both children and adolescents made judgments based on the characteristics of the bullying target) supports the assumption that social judgments of group exclusion are multi-dimensional [26,28]. In addition, the tendency for adolescents to judge peer exclusion positively was observed not only in Western cultures but also in Japan. These findings are of interest to researchers of bullying and children’s intergroup relationships. Although there were limitations to this study, such as the inability to determine why the judgments did not differ by bystander type and the method used to rate self-reported bullying behavior, the current study provides a foundation for future research on bullying.

## Figures and Tables

**Table 1:** Mean (SD) scores for judgment and emotion attribution by grade, condition, and type of target.

			4th (*n* = 110)		
			Boys	Girls	Total
Scores of peer exclusion judgment (a)					
	Excluded violent target story				
		No bystander condition	3.33 (0.82)	3.13 (0.92)	3.23 (0.86)
		Passive bystander condition	3.48 (0.77)	3.36 (0.73)	3.43 (0.74)
		Active bystander condition	3.75 (0.58)	3.63 (0.81)	3.69 (0.69)
	Excluded shy target story				
		No bystander condition	3.53 (0.92)	3.93 (0.26)	3.73 (0.69)
		Passive bystander condition	3.72 (0.46)	3.68 (0.57)	3.70 (0.51)
		Active bystander condition	3.75 (0.78)	3.81 (0.54)	3.78 (0.66)
Scores of emotion attributions (b)					
	Excluded violent target story				
		No bystander condition	2.93 (1.21)	2.80 (0.78)	2.86 (0.99)
		Passive bystander condition	2.91 (0.95)	2.70 (0.97)	2.80 (0.96)
		Active bystander condition	2.33 (0.98)	2.75 (1.13)	2.55 (1.06)
	Excluded shy target story				
		No bystander condition	2.64 (1.22)	2.93 (1.16)	2.79 (1.18)
		Passive bystander condition	2.91 (1.00)	2.35 (0.98)	2.63 (1.02)
		Active bystander condition	2.27 (0.96)	2.69 (1.20)	2.48 (1.09)
			**6th (*n* = 143)**		
			**boys**	**girls**	**total**
Scores of peer exclusion judgment					
	Excluded violent target story				
		No bystander condition	3.39 (0.70)	3.52 (0.59)	3.47 (0.63)
		Passive bystander condition	3.32 (0.85)	3.46 (0.76)	3.39 (0.80)
		Active bystander condition	3.42 (0.90)	3.13 (0.82)	3.29 (0.87)
	Excluded shy target story				
		No bystander condition	3.89 (0.32)	3.84 (0.37)	3.86 (0.35)
		Passive bystander condition	3.72 (0.46)	3.81 (0.49)	3.76 (0.47)
		Active bystander condition	3.88 (0.33)	3.70 (0.56)	3.80 (0.46)
Scores of emotion attributions					
	Excluded violent target story				
		No bystander condition	2.78 (1.06)	2.72 (0.98)	2.74 (1.00)
		Passive bystander condition	2.56 (0.92)	2.69 (0.68)	2.63 (0.80)
		Active bystander condition	2.58 (0.86)	2.35 (0.71)	2.47 (0.79)
	Excluded shy target story				
		No bystander condition	2.89 (1.02)	2.68 (0.90)	2.77 (0.95)
		Passive bystander condition	2.56 (0.96)	2.58 (0.70)	2.57 (0.83)
		Active bystander condition	2.58 (0.95)	2.57 (0.79)	2.57 (0.87)
			**8th (*n* = 118)**		
			**boys**	**girls**	**total**
Scores of peer exclusion judgment					
	Excluded violent target story				
		No bystander condition	2.73 (0.77)	2.43 (0.76)	2.61 (0.77)
		Passive bystander condition	2.69 (0.93)	3.35 (0.86)	2.95 (0.95)
		Active bystander condition	3.00 (0.65)	2.89 (0.90)	2.95 (0.77)
	Excluded shy target story				
		No bystander condition	3.50 (0.74)	3.57 (0.65)	3.53 (0.70)
		Passive bystander condition	3.50 (0.76)	3.82 (0.39)	3.63 (0.66)
		Active bystander condition	3.40 (0.75)	3.61 (0.70)	3.50 (0.73)
Scores of emotion attributions					
	Excluded violent target story				
		No bystander condition	2.05 (0.83)	2.29 (0.83)	2.15 (0.82)
		Passive bystander condition	1.92 (0.56)	2.29 (0.92)	2.07 (0.74)
		Active bystander condition	2.80 (0.89)	2.11 (0.76)	2.47 (0.89)
	Excluded shy target story				
		No bystander condition	2.20 (1.06)	2.64 (0.84)	2.38 (0.99)
		Passive bystander condition	2.15 (0.93)	2.47 (0.80)	2.28 (0.88)
		Active bystander condition	2.70 (0.92)	1.94 (0.73)	2.34 (0.91)

Note： (a) The higher the score, the higher the tendency to judge the exclusion as “wrong”. (b) The higher the score, the higher the tendency to attribute a “sad” feeling.

**Table 2:** Mean (SD) scores for bullying and bystander behavior by grade.

	4th		
	Boys	Girls	Total
Bullying behavior scale	0.74 (0.71)	0.72 (072)	0.73 (0.71)
Bystander behavior scale	0.60 (0.70)	0.42 (0.60)	0.51 (0.66)
	6th		
	boys	girls	total
Bullying behavior scale	0.91 (0.64)	0.62 (0.60)	0.76 (0.64)
Bystander behavior scale	0.64 (0.72)	0.60 (0.68)	0.62 (0.70)
	8th		
	boys	girls	total
Bullying behavior scale	0.96 (0.71)	0.67 (0.52)	0.84 (0.65)
Bystander behavior scale	0.83 (0.79)	0.75 (0.85)	0.79 (0.81)

**Table 3:** Hierarchical multiple regression analyses predicting bullying behaviors.

Predictor		R^2^		β	
Step 1		0.03	*		
	Grade			0.04	
	Gender			0.16	**
	No bystander condition			0.02	
	Passive bystander condition			0.06	
Step 2		0.08	***		
	Grade			−0.02	
	Gender			0.16	**
	No bystander condition			0.00	
	Passive bystander condition			0.06	
	Judgments toward the violent target			−0.23	***
	Judgments toward the shy target			−0.04	
Step 3		0.09	***		
	Grade			−0.03	
	Gender			0.16	**
	No bystander condition			0.00	
	Passive bystander condition			0.06	
	Judgments toward the violent target			−0.22	***
	Judgments toward the shy target			−0.03	
	Emotion attributions of violent target			−0.06	
	Emotion attributions of shy target			−0.03	
Total R^2^		0.09	**		

Note. * *p* < 0.05, ** *p* < 0.01, *** *p* < 0.001.

**Table 4:** Hierarchical multiple regression analyses predicting bystander behaviors.

Predictor		R^2^		β	
Step 1		0.05	**		
	Grade			0.17	
	Gender			0.06	
	No bystander condition			0.12	
	Passive bystander condition			0.10	
Step 2		0.09	***		
	Grade			0.12	
	Gender			0.05	
	No bystander condition			0.11	
	Passive bystander condition			0.10	
	Judgments toward the violent target			−0.16	**
	Judgments toward the shy target			−0.11	*
Step 3		0.09	***		
	Grade			0.10	
	Gender			0.05	
	No bystander condition			0.11	
	Passive bystander condition			0.11	
	Judgments toward the violent target			−0.15	**
	Judgments toward the shy target			−0.10	
	Emotion attributions of the violent target			−0.06	
	Emotion attributions of the shy target			−0.02	
Total R^2^		0.09	***		

Note. * *p* < 0.05, ** *p* < 0.01, *** *p* < 0.001

## Data Availability

Data sharing is not applicable due to some participants not agreeing to make the data public.

## Appendix A

Story of the excluded violent target:

Aki sometimes gets mad, hits other children, and gets violent. Makoto’s group, which is in the same class as Aki, has been annoyed with Aki. Aki repeatedly told Makoto and others, “I want to play with you,” but they refused, saying, “You cannot join our group. We don’t want to play with you.”

Story of the excluded shy target:

Haru is quiet and not good at talking with other children. Kaoru’s group, which is in the same class as Haru, thinks that it is not fun to play with Haru. Haru repeatedly told Kaoru and others, “I want to play with you,” but they refused, saying, “You cannot join our group. We don’t want to play with you.”

No bystander condition: The above sentences were presented.

The passive bystander condition included the following sentence: (Yu or Ritsu)’s group, which is in the same class, is watching the conversation between (Aki or Haru) and (Makoto or Kaoru) and others.

The active bystander condition included the following sentence: (Yu or Ritsu)’s group, which is in the same class, was watching the conversation between (Aki or Haru) and (Makoto or Kaoru) and other children and spoke to (Aki or Haru), saying, “Let’s play together.”
